# 3D motion capture data into a kinematic composite score for assessing musculoskeletal impairments

**DOI:** 10.1016/j.jbiomech.2025.112725

**Published:** 2025-04-26

**Authors:** Erin Archibeck, Ryan Halvorson, Pavlos Silvestros, Abel Torres-Espin, Grace O’Connell, Jeannie Bailey

**Affiliations:** a Department of Mechanical Engineering, University of California, Berkeley, USA; b Department of Orthopedic Surgery, University of California, San Francisco, USA; c School of Public Health Sciences, University of Waterloo, Waterloo, Canada; d Faculty of Rehabilitation Medicine, University of Alberta, Edmonton, Canada; e Department of Neurological Surgery, University of California, San Francisco, USA

## Abstract

Biomechanical analysis is essential for understanding and monitoring musculoskeletal impairments, with implications for clinical diagnostics and research. Current clinical methods provide isolated joint measures or qualitative observations, failing to capture motion complexity. While 3D biomechanical testing is comprehensive, its application is hindered by data volume, making it challenging to derive clinically relevant conclusions. Approaches to distill motion often neglect time-series data or are dependent on population size. To address these gaps, this study introduces the Kinematic Composite Score (K-Score), a metric that distills high-dimensional motion while preserving individual variability.

The objective of this research is to outline the methodology of the K-Score algorithm, highlight its strengths, limitations, and applications. We conducted a comparative study of the K-Score Algorithm against (1) the conventional isolated kinematic measures, and (2) traditional Principal Component Analysis. The analysis was conducted with a cohort of chronic low back pain (LBP) patients, who exhibit tremendous movement heterogeneity.

The K-Score outperformed traditional isolated metrics in differentiating overall motion of LBP patients from healthy controls (K-Score: controls = 94.16 ± 2.64, LBP = 85.82 ± 7.73, p < 0.001). The K-Score also demonstrated significant differences in overall motion between male and female participants, where females with LBP demonstrated higher scores than males (p < 0.001). Importantly, the K-Score was not sensitive to BMI (p = 0.49), age (p = 0.14), height (p = 0.11), or sample size. In conclusion, the K-Score addresses key limitations of traditional approaches by encapsulating full-body, time-series data within a single score that is adaptable across motion capture systems and activities, making it a powerful tool for clinical biomechanics research.

## 1. Introduction

Musculoskeletal (MSK) conditions are the leading global contributor to chronic pain and disability, primarily characterized by biomechanical impairment (Blyth et al., 2019; GBD 2016 DALYs and HALE Collaborators, 2017; Piedrahita, 2006). Biomechanical analysis is crucial for identifying abnormal motions that may be caused by or contribute to MSK pathology. Beyond a standard physical examination, clinical tools for measuring biomechanical function include 1) static goniometry (Gajdosik & Bohannon, 1987; Laupattarakasem et al., 1990; van Trijffel et al., 2010), 2) functional testing (de Melo et al., 2022; Kahraman et al., 2016; M. R. Pourahmadi et al., 2018), and 3D skeletal tracking (Lam et al., 2023; do Rosário, 2014). Although useful historically and in some specific situations, static goniometry and functional testing generally fail to capture the complexity of full-body motion and require specialized training, which can affect reliability (van Trijffel et al., 2010) and lack consistency (Finley et al., 2015; Norkin & White, 2016; Reissner et al., 2019). In contrast, contemporary 3D biomechanical analysis leads to a large dataset consisting of three-dimensional data for every landmark across time, highlighting the necessity to extract meaningful and clinically useful metrics.

Traditionally, 3D skeletal tracking data is analyzed by either (1) extracting isolated kinematic/kinetic measures, often using biomechanical models, or (2) reducing the dimensionality of the data to create a single metric, often through Principal Component Analysis (PCA). Isolated kinematic measures are frequently selected from the dataset, but determining whether these measures effectively capture a patient’s overall biomechanical impairment is challenging. For example, in chronic low back pain (LBP) patients, the highest prevalent MSK condition globally (Wu et al., 2020), lumbar range of motion and higher order trunk kinematics (maximum velocity, acceleration) are frequently studied (Laird et al., 2014; Lehman, 2004; Poitras et al., 2000). However, these measures exhibit high variability, conflicting results, neglect time-series data, and overlook meaningful full-body (including lower extremity) compensatory strategies (McGregor et al., 1997; Papi et al., 2018; Song et al., 2012). Their usefulness may be limited to specific patients, activities, or time points, whereas growing evidence supports a full-body approach for a more comprehensive assessment of biomechanical function (Papi et al., 2018).

On the other hand, kinematic scores aimed to condense 3D biomechanical data into a single metric have been developed for gait (Herrera-Valenzuela et al., 2022; Massaad et al., 2014; Schwartz & Rozumalski, 2008), balance (Chang et al., 2020; Eveleigh et al., 2023; Halvorson et al., 2022), and upper extremity motion (Jurkojć et al., 2017). Further, many scores highlight deviations from healthy controls, suggesting promising clinical utility. Although these scores provide a valuable foundation for our work, they also present several limitations, including one or more of the following: lack of compatibility with other activities, insufficient coverage of full-body posture, lack of dynamic analysis by neglecting time series data, and dependence on patient sample size. Due to the volume of motion capture data, PCA is widely used for dimensionality reduction and calculating these kinematic scores. PCA transforms original data into a set of uncorrelated components that capture the most significant variance, highlighting meaningful patterns within datasets. PCA is typically employed across patients over kinematic measurements at select or single time points (Brandon et al., 2013; Deluzio & Astephen, 2007; Halvorson et al., 2022; Keller et al., 2022; Warmenhoven et al., 2021). Although this approach has shown to be useful in a research context, it challenges clinical utility as applying PCA across patients conflicts patient-specific movements with global population movement patterns that might not reflect any individual subject. An additional challenge with across-patient PCA and other machine-learning techniques is the requirement for large sample sizes to compute a stable metric. To address these limitations, PCA can be applied at the individual level, as previously demonstrated in other fields and some kinematic analyses [28]. However, existing research in kinematics primarily focuses on describing motion patterns rather than developing a composite metric for clinical utility.

Therefore, in line with a more personalized assessment of MSK impairments, the objective of this study was to detail the methodology of the K-Score algorithm and evaluate its strengths and limitations. We aimed to explore its potential applications as a quantitative approach for assessing differences in postural movement patterns, defined by alignment with healthy control motion, across multiple body landmarks and time. Posture reflects the spatial relationship of the body, is a primary mode of compensation, and is fundamental to understanding differences in biomechanical function and motor control (Park et al., 2023; M. Pourahmadi et al., 2023; Sung & Lee, 2024). Further, the K-Score addresses the challenges of current approaches by capturing full-body motion that can be used across diverse activities, incorporating dynamic time-series data, and providing a comprehensive, single metric that does not depend on patient sample size, ensuring potentially broad applicability for both clinical and research contexts. We conducted a comparative analysis to evaluate the advantages and limitations of the K-Score algorithm in relation to traditional isolated kinematic metrics and across-patient PCA analysis. All three approaches were applied to identify differences within patients with chronic LBP, which was selected due to its high prevalence (Wu et al., 2020) and tremendous heterogeneity in movement patterns (Alsubaie et al., 2023).

We hypothesized that the K-Score metric would effectively capture the coordinated interactions across multiple body segments over time that quantify differences in overall movement patterns, while maintaining independence from sample size and demonstrating robustness against demographic variability. This would make it more effective at distinguishing patient types and reducing the limitations typically associated with traditional isolated metrics and the conventional PCA method. In both clinical and research settings, the K-Score could be applied to evaluate movement impairments across different patient populations, monitor treatment effectiveness and recovery, and identify movement adaptations that contribute to long-term MSK health.

## 2. Methods

### 2.1. Subject populations

Our institutional review board approved all study activities and informed consent was obtained from all participants before data collection (lRB #2031485, #1621015, #20204648). Data was obtained from patients with LBP (n = 317) and a cohort of age and sex-matched controls (CTRL, n = 62). LBP patients were selected from the Longitudinal Clinical Cohort for Comprehensive Deep Phenotyping of Chronic Low-Back Pain Adults Study, also called the Comeback cohort. Patients with LBP for more than 3 months and > 50 % of days were included in the study and age-matched CTRL participants were a normative measure for the biomechanical analysis. In the LBP group, 55 % of participants were female (LBP-F; n = 174; age = 56 ± 12 years, BMI = 26.9 ± 6.7), and 45 % were male (LBP-M; n = 143; age = 56 ± 13years, BMI = 26.5 ± 5.9). Exclusion criteria for all groups included: any contraindications to MRI; history of discitis, osteomyelitis, spine tumor, ankylosing spondylitis, rheumatoid arthritis, polymyalgia rheumatica, psoriatic arthritis, or lupus; history of any bone-related cancer or cancer that metastasized to the bone; current cancer treatment or plans to begin treatment within the next 12 months; any cancer treatment in the past 24 months; vertebral fracture within the last 6 months; history of cauda equina syndrome or severe leg weakness (e.g., foot drop) due to a low back condition; referral pain; BMI > 35; and inability to walk unaided.

### 2.2. Markerless motion capture assessments

**Fig. 1.**
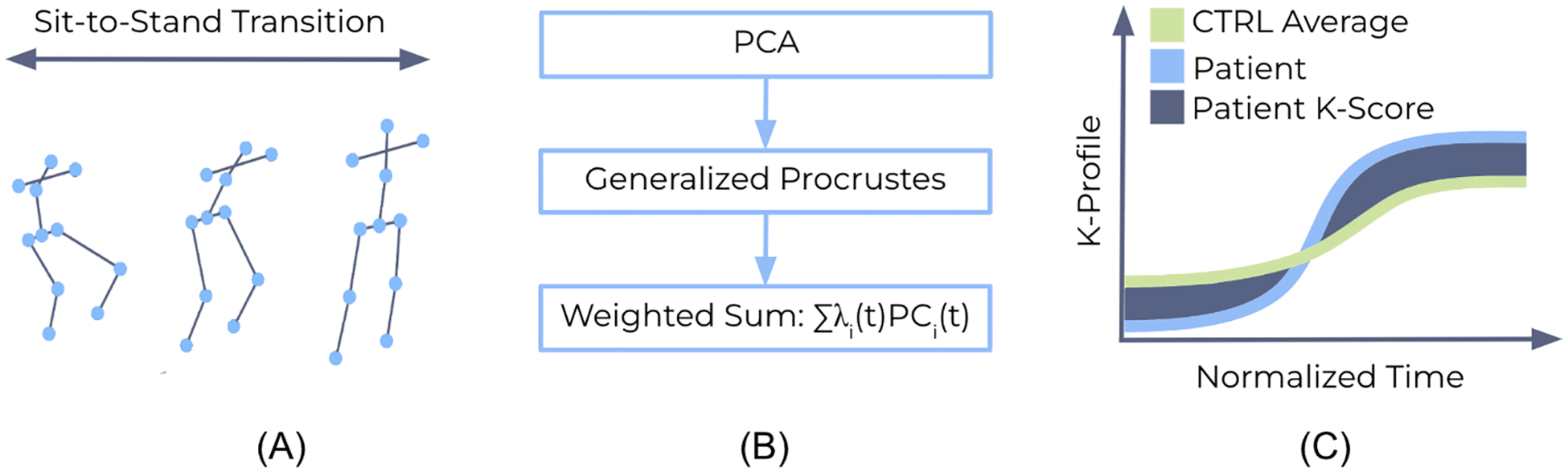
(A) Step 1: Capture landmark positions during the sit-to-stand transition (B) Step 2: Input positional landmarks across time into PCA, apply generalized Procrustes across patients, and calculate the weighted sum for each individual (C) Step 3: Generate the Kinematic Profile and Kinematic Composite Score.

A full-body markerless motion capture system (Azure Kinect, Microsoft) was employed to estimate joint position (sampling frequency rate = 30 Hz) during sit-to-stand (STS). STS is a relevant functional task characterized by full-body involvement of the trunk and lower extremities (de Melo et al., 2022; Özüdoğru et al., 2023). The Kinect depth mapping camera was positioned two meters in front of the subject at waist height, and the system was calibrated prior to data collection to ensure the participant was fully within the camera’s view, with an uncluttered background and unobstructed joints. Eleven landmarks from the markerless skeletal tracking output were used, including five on the trunk (neck, left and right shoulders, mid-spine, and the base of the spine) and six on the lower extremities (left and right hips, knees, and ankles) (Fig. 1A). For the five STS trials, participants performed the task using a standard 17-inch height chair with arms by their side and feet placed hip-width apart. Participants were instructed to move at a natural and comfortable pace, with timed audio cues provided between each sit-to-stand trial.

### 2.3. Kinematic composite score algorithm

The raw 3D skeletal tracking data was filtered using a bidirectional 5 Hz second-order low-pass Butterworth filter, and biomechanical constraints were applied to improve measurements and reduce mean absolute errors and intraclass correlation coefficients (Matthew et al., 2019). For STS, the five repetitions were separated to highlight the dynamic transition period, defined as the motion from a seated to a standing position, using a threshold-based peak detection custom algorithm. Repetitions 2–5 were analyzed, while the first repetition was removed due to significant inconsistencies identified in the preliminary analysis. The data was temporally normalized using min–max scaling, rescaling the time values to a range of 0 to 100 % by subtracting the minimum and dividing by the range.

**Fig. 2.**
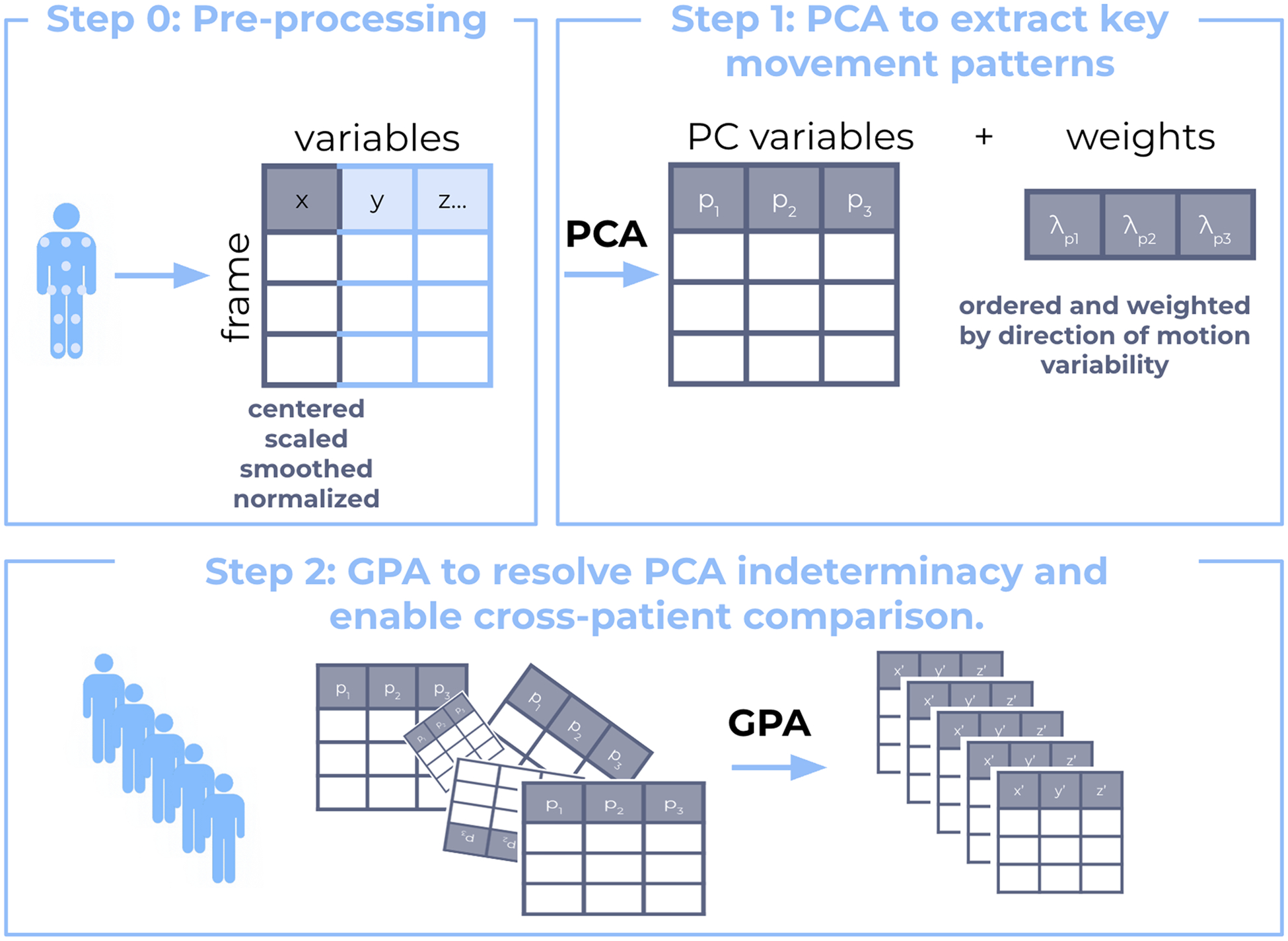
Schematic of Kinematic Composite Score Algorithm to highlight Step (0): pre-processing, Step (1): Apply PCA to extract key movement patterns, and step (2): Apply GPA to align values and enable cross-patient comparison.

Using the Python scikit-learn library (Sklearn.Decomposition.PCA, n. d.), PCA was applied to $X_{i}$, a TxJ matrix for patient i, where T is the number of time points and J is the number of body landmarks (Equation 1). $X_{i}$ is a filtered, segmented, and time-normalized positional data matrix for each maneuver. Before computing PCA, each joint trajectory was centered to remove the mean trajectory over time and standardized to have zero mean and unit variance, ensuring that variability is not dominated by differences in absolute magnitude (Fig. 2). To perform PCA on $X_{i}$, the covariance matrix was calculated to determine the eigenvalues and eigenvectors. The resulting transformed data is $P_{i}$, a TXK matrix, where K is the number of principal components (PC) retained. For this calculation, K = J, as all PC values were employed. $W_{i}$ is equal to the PC loadings, with a size equal to JxK (Equation 2).

[1] $$X_{i}=\left(\begin{array}{cccc} x_{11} & x_{12} & \cdots & x_{1J}\\ x_{11} & x_{22} & \cdots & x_{2J}\\ \vdots & \vdots & \ddots & \vdots \\ x_{T1} & x_{T2} & \cdots & x_{TJ} \end{array}\right)$$

[2] $$P_{i}=X_{i}W_{i}$$

While PCA finds the direction of motion variability for each subject, Generalized Procrustes Analysis ensures that all subjects’ motion patterns are aligned in shape space by removing residual differences in translation, rotation, and scale (Torres-Espin et al., 2024), removing the need for individual anthropometric measurements. Therefore, GPA was applied to the PCA-transformed dataset across all patient types for cross-subject comparisons. GPA aligns all PC shapes according to a reference frame, which was selected as the “resting position” (t = 0) for the healthy control average, where PC_i_ is the Procrustes-transformed PC score of the i-th patient and s,r, and t are the scaling, rotation, and translation factors, respectively (Equation 3).

[3] $$PC_{i}=s_{i}r_{i}P_{i}+t_{i}$$

The weighted sum of the PC scores at each time point was calculated using the corresponding eigenvalues as weights. Each weighted sum was defined as a point on the Kinematic Profile (K-Profile), where $\lambda_{\text{i},\text{k}}$ represents the eigenvalue and ${\text{PC}}_{\text{i},\text{k}}(t)$ represents the score of the i-th patient and the k-th PC at time t (Equation 4, Fig. 1B).

[4] $${\text{KProfile}}_{i}(t)=\sum_{k=1}^{K}\lambda_{i,k}(t)PC_{i,k}(t)$$

To quantify this curve, the Kinematic Composite Score (K-Score) was developed by measuring a “deviation factor”, DF, which integrates the absolute difference between the individual’s and the control average’s K-Profile (Equation 5, Fig. 1C). DF measures the total alignment to the “ideal trajectory”, defined as the average of the healthy control group with no reported pain or biomechanical impairment.

[5] $$DF_{i}=\int_{t=0}^{100}\left|{\text{KProfile}}_{i}(t)-\text{average}\left({\text{KProfile}}_{\text{control}}(t)\right)\right|dt$$

To account for patient speed, DF can be adjusted by multiplying the ratio of the individual’s and control group’s average time (T). The K-Score values are transformed to enhance comprehensibility for clinicians and patients, such that 100 represents the control average movement trajectory. To do so, the magnitude of the time-incorporated deviation factor is adjusted by a scaling factor, α and subtracted from 100 (Equation 6).

[6] $${\text{KScore}}_{i}=100-\frac{1}{\alpha }\left(\frac{T_{i}}{\text{average}\left(T_{\text{control}}\right)}\right)DF_{i}$$

### 2.4. Traditional isolated metrics

Several traditional torso-related traditional metrics were quantified (Matthew et al., 2019) for comparison to the K-Score. The maximum torso flexion angle (degrees) was determined by calculating the angle between the torso body segment axis and the world vertical axis in the sagittal plane, identifying the peak flexion value during sit-to-stand movement. Peak Sagittal Vertical Alignment (SVA) was measured as the maximum anterior displacement of the shoulder joint relative to the hip joint and normalized by subject height. The maximum torso velocity was computed in the anterior (horizontal) and superior (vertical) directions by differentiating the torso’s position (center of mass) over time and identifying the peak velocity in each direction. Velocity was normalized by the square root of the product between gravitational acceleration and the individual’s leg length. Similarly, the maximum torso acceleration was determined in the anterior (horizontal) and superior (vertical) directions by differentiating the torso’s velocity and identifying the peak acceleration in each direction, normalized by gravitational acceleration. As a result, all measures are unitless.

### 2.5. Traditional PCA approach

For each subject, a (1 × T•J) vector was constructed, where T represents the number of time points and J denotes the number of body landmarks, following the methodology of the K-Score Algorithm. To minimize variations in initial positioning, orientation, and scale, GPA was applied to the raw body landmark data before vectorization. A data matrix of dimensions (M•I) × (T•J) was then assembled, where M corresponds to the number of trials and I to the number of subjects. Prior to performing PCA, the data was mean-centered and standardized. PCA was performed on the dataset, and PC scores for each participant were analyzed, with 5 PC scores evaluated to retain 90 % of the variance in the data.

### 2.6. Statistical analysis

To evaluate how well each measure (K-Score, traditional isolated metrics, and PC values) differentiates between the CTRL and LBP groups, Kruskal-Wallis tests (scipy.stats.kruskal) were employed due to the nonparametric nature of the data, as confirmed by the Shapiro-Wilk Normality Test (scipy.stats.shapiro). To measure effect size, Rank-Biserial correlation (r_s_) was calculated by assessing the rank differences between the CTRL and LBP groups. Variability was assessed using the robust coefficient of variation (CV), calculated as the median absolute deviation divided by the median, multiplied by 100.

Additionally, the relationships between these variables and demographic factors (age, sex, BMI, and height) were analyzed. Continuous variables (age, BMI, and height) were examined using linear regression (sklearn.linear_model.LinearRegression), with R2 and p-values reported. Sex differences were assessed using the Kruskal-Wallis test, and if significant, Dunn’s post hoc analysis (scipy.stats, statsmodels. stats.multitest) was conducted for pairwise comparisons. A significance threshold of p < 0.05 was used. All variables were plotted and reported as median ± interquartile range (IQR).

## 3. Results

### 3.1. Interpretation of K-Profile & K-Score

**Fig. 3.**
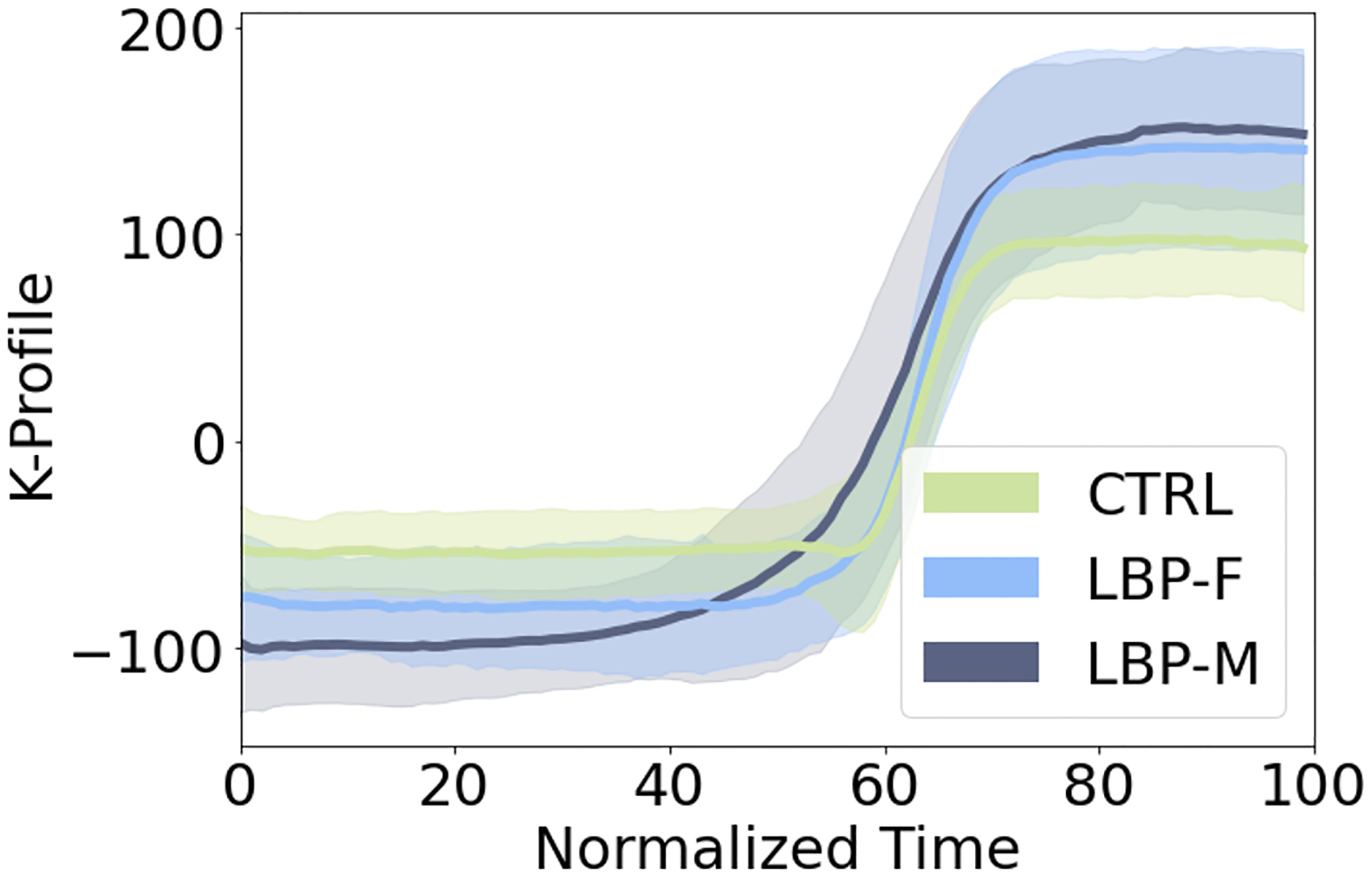
K-Profiles for CTRLs, LBP-F, and LBP-M plotted as median ± IQR for the K-Score Algorithm.

For each individual, the K-Profile, a dimensionless time-series curve, captures the most prominent postural patterns across all landmarks over time, providing a valuable visual tool for analyzing motion trajectories (Fig. 3). In contrast, the selected isolated metrics do not capture the capacity of full-body time-series data. Likewise, the traditional PCA approach lacks a motion trajectory, as time is embedded within the vectorized representation before PCA, resulting in a single score per motion. To assess the individual movement patterns quantitatively, the K-Score calculates the total difference in the individual’s K–profile compared to the healthy control average, yielding a meaningful value that measures how much an individual patient deviates from healthy motion. Conversely, isolated kinematic measures and traditional PC scores fail to inherently provide a value that can be easily interpreted and reflect deviations in movement patterns relative to a baseline.

### 3.2. Descriptive statistics and group differentiation

**Fig. 4.**
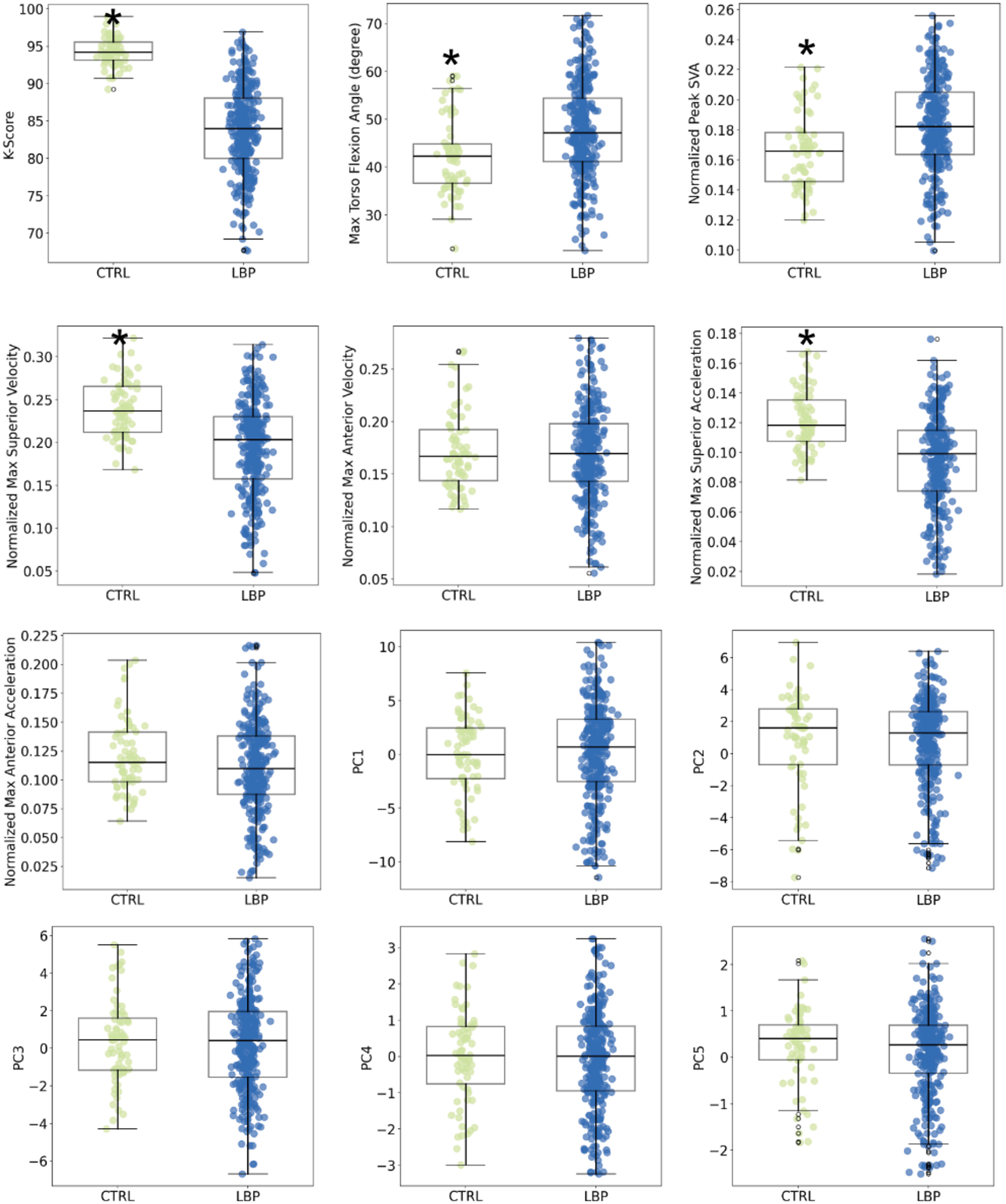
K-Scores for CTRL and LBP patients plotted as median ± IQR for K-Score, Traditional Isolated Metrics, and PC1–5. * indicates p < 0.05 for pairwise comparison to CTRL group.

When comparing the K-Score to traditional measures, the K-Score exhibited the most pronounced difference between the healthy control (CTRL) and low back pain (LBP) groups (r_s_ = 0.83), with the control group (94.16 ± 2.64) revealing significant higher scores than the LBP group (85.82 ± 7.73, p < 0.001) (Table 1, Fig. 4). Other variables, including maximum torso flexion angle, peak SVA, maximum torso superior velocity, and maximum torso superior acceleration, validated the significant difference in motion between the CTRL and LBP groups (p < 0.001). However, their effect sizes (−0.35, −0.35, 0.49, and 0.51, respectively) were smaller than that of the K-Score, indicating a weaker measure of the difference between the two groups. Additionally, the K-Score exhibited the lowest variability among the CTRL group and the second-lowest variability among the LBP group, highlighting its reliability as a measure of movement patterns. None of the 5 PC scores showed a significant difference between the CTRL and LBP groups.

### 3.3. Associations with demographic factors

The K-Score showed no significant relationships with BMI, age, or height (p > 0.1). While the traditional isolated metrics were not associated with BMI or age, all but the max superior acceleration showed weak but significant associations with height (p < 0.03). PC2–PC4 were also associated with height (p < 0.04), PC1 was significantly associated with BMI (p = 0.008), and PC3 was associated with age (p < 0.001).

**Fig. 5.**
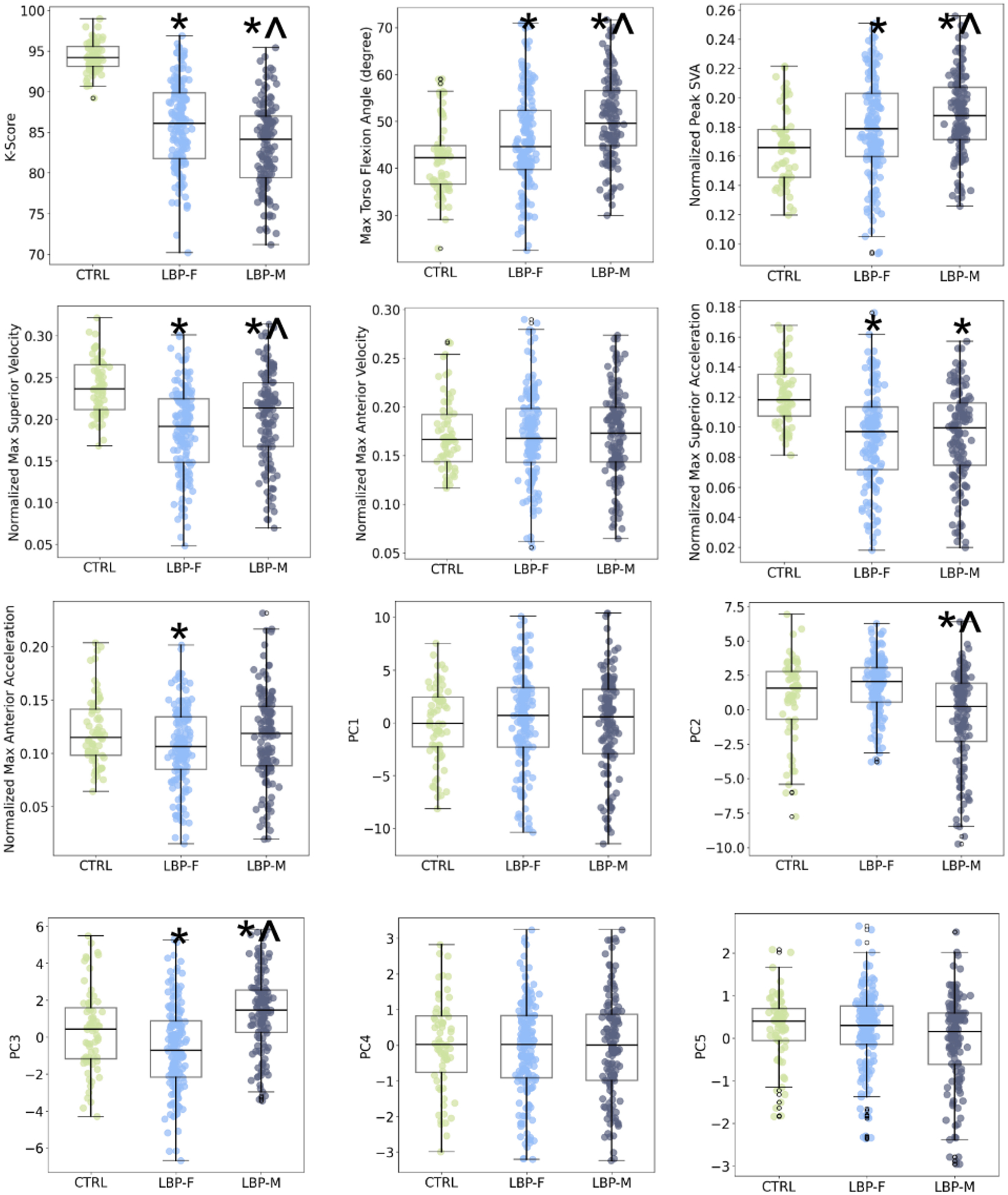
K-Scores for CTRL, LBP-F, and LBP-M patients plotted as median ± IQR for K-Score, Traditional Isolated Metrics, and PC1–5. * indicates p < 0.05 for pairwise comparison to CTRL group and^indicates p < 0.001 for pairwise comparison to LBP-F group.

Sex differences were also examined to determine if the groups had sufficient granularity to detect differences in postural movement patterns based on sex (Table 2, Fig. 5). The LBP-F and LBP-M groups were matched for age and BMI (p = 0.77, 0.92, respectively). Significant differences were observed between males and females in K-Scores (p < 0.001), Torso Flexion Angle (p = 0.003), Superior Velocity (p = 0.005), Anterior Velocity (p = 0.05), Anterior Acceleration (p = 0.03), and PC2 & PC3 (p < 0.001).

### 3.4. Sample size independence analysis

**Fig. 6.**
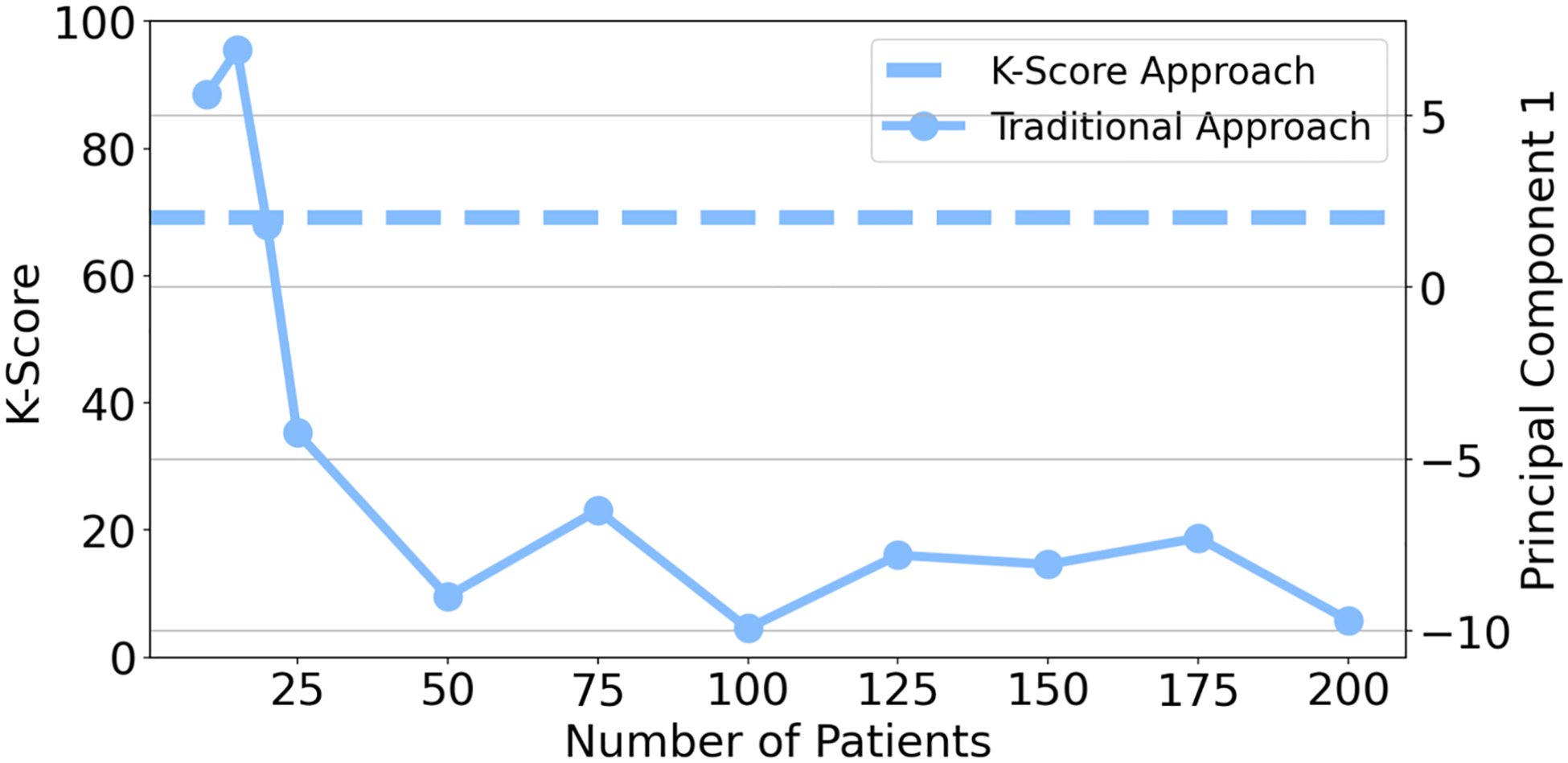
The K-Score and Principal Component 1 values plotted across a varying study sample size for a randomly selected LBP patient.

The K-Score and PC1 values of a randomly selected LBP patient was plotted across various study sample sizes (Fig. 6). While the PC scores exhibited high variability, the K-Score Algorithm produced a constant metric regardless of sample size.

## 4. Discussion

We developed a method for distilling within-subject skeletal movement into a composite metric for clinical research utility. A comparative analysis was applied to illustrate the potential advantages and disadvantages of our K-Score metric, studying patient-specific motion, in comparison to traditional isolated kinematic measures and the conventional population-based PCA approach. It is important to note that while the K-Score itself does not provide measures of specific differences in postural movement patterns, it facilitates deeper analysis of movement distinctions by examining the underlying PC scores at each time point. However, the primary use case is not to assess each individual body movement but to provide a comprehensive and robust measure of overall postural movement patterns. This enables comparisons within a single subject, within groups, and between groups. This metric can be used to better understand how patient biomechanical impairment may relate to treatment outcomes and factors, such as pain (Archibeck et al., 2025) and muscle quality (Halvorson & Archibeck, 2025).

Previous research has identified biomechanical differences between LBP and controls, as well as sex-based differences within LBP (Gombatto et al., 2006; Hoffman et al., 2012; Rahimi, 2020; Scholtes & Van Dillen, 2007), but the findings are inconsistent and challenging to consolidate due to the wide range of metrics, landmarks, and measurement protocols. K-Score addresses this by consolidating the critical movement patterns and deviations, providing a clear and consistent way to highlight key differences. The K-Score demonstrated the most pronounced differences in distinguishing LBP patients from healthy controls (rs = 0.83), with the control group showing significantly higher scores (94.16 ± 2.64) compared to the LBP group (85.82 ± 7.73, p < 0.001). While other traditional metrics (maximum torso flexion angle, peak SVA, max superior velocity, max superior acceleration), also identified significant differences between the groups, their smaller effect sizes (ranging from −0.35 to 0.51) indicated weaker measures. K-Score also demonstrated the sensitivity to sex-based differences in movement within the LBP group (p < 0.001 for K-Score), uncovering that females exhibit movement that aligns more to healthy controls compared to males. Other traditional metrics, such as flexion angle, peak SVA, anterior acceleration, and PC2 and PC3, show similar trends, indicating that females move more similarly to controls than males. This consistency further validates the K-Score, underlining its ability to integrate multiple aspects of individual isolated metrics by incorporating multiple degrees of freedom, time-series data, and inter-segmental relationships to highlight the key differences.

Furthermore, the K-Score is a robust approach, not influenced by BMI, age, height, or patient sample size. While the traditional isolated metrics were not significantly associated with BMI or age, five out of six still showed weak but significant associations with height, even after normalization. Despite attempts to adjust for body size, height remains a significant source of variability in these isolated metrics. Further, by using individual-based PCA to calculate the K-Score, not only are the full-body and temporal characteristics of each participant preserved, but it also ensures the scores are independent of sample size. In contrast, the traditional PCA (PC1-PC5), as used in other kinematic scores (Eveleigh et al., 2023; Halvorson et al., 2022), shows considerable metric variation for each patient depending on the number of patients included in the analysis (Fig. 6).

The K-Profile and K-Score has clinical and research utility. When compared to the traditional PCA, isolated kinematic metrics, and other kinematic scores (Jurkojć et al., 2017; Schwartz & Rozumalski, 2008), the ability to compute a full-body motion trajectory over time (K-Profile) provides valuable insight for both research and clinical applications (Fig. 3). For example, this has utility for leveraging K-Profiles to determine *when* patients exhibit compensatory strategies (relative to health control K-Profiles) and whether these moments temporally align with reported instances of movement-evoked pain or moments of mechanical dysfunction. Along with the K-Profile, having a K-Score metric allows for direct comparisons with other patient factors. For example, the K-Score provides deeper insight into how sex-based movement variability in LBP patients correlates with pain measures, including patient-reported outcomes and pressure pain threshold (PPT) (Archibeck et al., 2025). Additionally, the K-Score has exhibited the granularity to differentiate underlying pain mechanisms (nociceptive vs. nociplastic) in LBP patients, allowing us to begin untangling the heterogeneous nature of LBP and ultimately provide patient-specific recommendations based on pain type. Finally, the K-Score has also been used to assess biomechanical function and quantify its association with imaging factors (such as muscle quality and arthritis severity) to identify risk factors for delayed functional recovery following total hip arthroplasty (Halvorson & Archibeck et al., 2025).

While the K-Score Algorithm improves upon many of the short-comings of traditional approaches, it still has several limitations. Primarily, due to the incorporation of time series into the analysis, the K-Score Algorithm is highly sensitive to data segmentation and must be done methodically and consistently. Further, any approach applying PCA assumes that the relationship between all variables (landmark positions) is linear. While the non-linear effects are minimal on simple and predictable tasks such as the sit-stand transition, the PCA assumption may not hold for more complex dynamic motions. The K-score is also designed to constrain variability in the controls. However, this may minimize the effect of confounding from age, height, and BMI. Finally, the algorithm is dependent on the control sample size, as direct comparisons are made to the control average. However, since variability within the healthy control group is minimal, a large control sample size may not be necessary. Additional validation studies with diverse musculoskeletal patient populations and activities are needed to confirm these findings’ effectiveness and broader applicability. Further, this methodology can be extended to quantify differences in body velocity and acceleration profiles over time by modifying the input parameters accordingly.

## 5. Conclusion

Employing a single kinematic outcome measure to quantify biomechanical function may not adequately capture the full-body compensatory strategies of each individual (Quirk et al., 2023). Traditional PCA, used in many kinematic metrics, is dependent on patient sample size and does not commonly output time-series data. Therefore, we created a composite metric, the K-Score, which captures the prominent patterns across all landmarks over time. The algorithm is adaptable for any motion capture technology that provides position-based data at a reasonable sampling rate, any landmark positions of interest, and any activity providing standardized conditions maintained across participants. The K-Score is an innovative approach that addresses several limitations in previous biomechanical composite metrics, including encompassing full-body posture, incorporating time series data, and studying subject-specific rather than population-based data.

## Figures and Tables

**Table 1:** Summary Statistics (Median, IQR, p-value, and coefficient of variation) for CTRL and LBP patients for K-Score, Traditional Isolated Metrics, and PC1–5.

Variable	Median (IQR)				CV%	
	Control	LBP	p-value	r_s_	Control	LBP
K-Score	94.16 (2.64)	85.82 (7.73)	<0.001	0.83	7.57	11.64
Max Torso Flexion Angle (degrees)	42.38 (9.58)	47.11 (13.34)	<0.001	−0.35	13.10	13.34
Normalized Peak SVA	0.17 (0.03)	0.18 (0.04)	<0.001	−0.35	11.93	11.34
Normalized Max Superior Velocity	0.24 (0.06)	0.20 (0.08)	<0.001	0.49	11.68	15.98
Normalized Max Anterior Velocity	0.17 (0.05)	0.17 (0.06)	0.90	−0.01	14.76	16.61
Normalized Max Superior Acceleration	0.12 (0.03)	0.10 (0.04)	<0.001	0.51	11.03	20.18
Normalized Max Anterior Acceleration	0.12 (0.04)	0.11 (0.05)	0.34	0.12	18.01	23.80
PC1	−0.05 (4.89)	0.69 (5.92)	0.52	−0.04	––	––
PC2	1.11 (4.19)	1.20 (3.89)	0.89	0.03	––	––
PC3	0.39 (2.77)	0.35 (3.53)	0.79	−0.01	––	––
PC4	0.02 (1.66)	0.02 (1.82)	0.66	0.04	––	––
PC5	0.33 (1.08)	0.21 (1.30)	0.56	0.07		

**Table 2:** Regression analyses of demographic factors (BMI, age, height, sex) on K-Score, Traditional Isolated Metrics, and PC1–5 for CTRL and LBP patients (${\text{R}}^{2}$, p-value).

	BMI (${\text{R}}^{2}$, p)	Age (${\text{R}}^{2}$, p)	Height (${\text{R}}^{2}$, p)	Sex(p)
K-Score	0.0015, 0.49	0.0069, 0.14	0.0084, 0.10	<0.001
Max Torso Flexion Angle (degrees)	0.0042, 0.26	0.0000, 0.97	0.0349, 0.001	0.003
Normalized Peak SVA	0.0032, 0.33	0.0007, 0.66	0.0291, 0.003	0.65
Normalized Max Superior Velocity	0.0001, 0.88	0.0057, 0.19	0.0273, 0.004	0.005
Normalized Max Anterior Velocity	0.0005, 0.70	0.0045, 0.25	0.0165, 0.03	0.05
Normalized Max Superior Acc.	0.0010, 0.58	0.0022, 0.41	0.0069, 0.15	0.73
Normalized Max Anterior Acc.	0.0034, 0.31	0.0021, 0.43	0.0411, <0.001	0.03
PC1	0.0224, 0.01	0.0032, 0.32	0.0072, 0.14	0.53
PC2	0.0000, 0.92	0.0062, 0.16	0.0270, 0.005	<0.001
PC3	0.0100, 0.08	0.0513, 0.00	0.2432, 0.005	<0.001
PC4	0.0003, 0.76	0.0031, 0.32	0.0149, 0.03	0.83
PC5	0.0107, 0.07	0.0001, 0.89	0.0027,0.36	0.39

## Data Availability

The data that support the findings of this study are maintained and archived at The UCSF Core Center for Patient-centric Mechanistic Phenotyping in Chronic Low Back Pain (UCSF REACH). Data and the K-Score code are available from the corresponding author, upon reasonable request, and with permission of UCSF REACH.
